# The Cerebral Cost of Breathing: An fMRI Case-Study in Congenital Central Hypoventilation Syndrome

**DOI:** 10.1371/journal.pone.0107850

**Published:** 2014-09-30

**Authors:** Mike Sharman, Cécile Gallea, Katia Lehongre, Damien Galanaud, Nathalie Nicolas, Thomas Similowski, Laurent Cohen, Christian Straus, Lionel Naccache

**Affiliations:** 1 Institut National de la Santé et de la Recherche Médicale (INSERM), Institut du Cerveau et de la Moelle Epinière (ICM), Unité Mixte de Recherche 1127, PICNIC Lab, Paris, France; 2 Centre National de la Recherche Scientifique (CNRS), Institut du Cerveau et de la Moelle Epinière (ICM), Unité 7225, PICNIC Lab, Paris, France; 3 Institut National de la Santé et de la Recherche Médicale (INSERM), Institut du Cerveau et de la Moelle Epinière (ICM), Unité Mixte de Recherche 1127, (CENIR), Paris, France; 4 Assistance Publique–Hôpitaux de Paris, Groupe hospitalier Pitié- Salpêtrière Charles Foix, Department of Neuroradiology, Paris, France; 5 Université Pierre et Marie Curie-Paris 6, Faculté de Médecine Pitié-Salpêtrière, Paris, France; 6 Assistance Publique–Hôpitaux de Paris, Groupe hospitalier Pitié- Salpêtrière Charles Foix, Centre d'Investigation Clinique 1421, Paris, France; 7 Assistance Publique–Hôpitaux de Paris, Groupe Hospitalier Pitié-Salpêtrière Charles Foix, Service de Pneumologie et Réanimation Médicale (Département “R3S”), Paris, France; 8 Sorbonne Universités, Université Pierre et Marie Curie-Paris 6, Unité Mixte de Recherche 1158 “Neurophysiologie Respiratoire Expérimentale et Clinique”, Paris, France; 9 Institut National de la Santé et de la Recherche Médicale (INSERM), Unité Mixte de Recherche 1158 “Neurophysiologie Respiratoire Expérimentale et Clinique”, Paris, France; 10 Assistance Publique–Hôpitaux de Paris, Groupe hospitalier Pitié-Salpêtrière Charles Foix, Department of Neurology, Paris, France; 11 Assistance Publique–Hôpitaux de Paris, Groupe Hospitalier Pitié-Salpêtrière Charles Foix, Service des Explorations Fonctionnelles de la Respiration, de l'Exercice et de la Dyspnée (Département “R3S”), Paris, France; 12 Assistance Publique–Hôpitaux de Paris, Groupe Hospitalier Pitié-Salpêtrière Charles Foix, Centre de Référence Maladies Rares “syndrome d'Ondine”, Paris, France; 13 Assistance Publique–Hôpitaux de Paris, Groupe hospitalier Pitié-Salpêtrière Charles Foix, Department of Neurophysiology, Paris, France; National Scientific and Technical Research Council (CONICET), Argentina

## Abstract

Certain motor activities - like walking or breathing - present the interesting property of proceeding either automatically or under voluntary control. In the case of breathing, brainstem structures located in the medulla are in charge of the automatic mode, whereas cortico-subcortical brain networks - including various frontal lobe areas - subtend the voluntary mode. We speculated that the involvement of cortical activity during voluntary breathing could impact both on the “resting state” pattern of cortical-subcortical connectivity, and on the recruitment of executive functions mediated by the frontal lobe. In order to test this prediction we explored a patient suffering from central congenital hypoventilation syndrome (CCHS), a very rare developmental condition secondary to brainstem dysfunction. Typically, CCHS patients demonstrate efficient cortically-controlled breathing while awake, but require mechanically-assisted ventilation during sleep to overcome the inability of brainstem structures to mediate automatic breathing. We used simultaneous EEG-fMRI recordings to compare patterns of brain activity between these two types of ventilation during wakefulness. As compared with spontaneous breathing (SB), mechanical ventilation (MV) restored the default mode network (DMN) associated with self-consciousness, mind-wandering, creativity and introspection in healthy subjects. SB on the other hand resulted in a specific increase of functional connectivity between brainstem and frontal lobe. Behaviorally, the patient was more efficient in cognitive tasks requiring executive control during MV than during SB, in agreement with her subjective reports in everyday life. Taken together our results provide insight into the cognitive and neural costs of spontaneous breathing in one CCHS patient, and suggest that MV during waking periods may free up frontal lobe resources, and make them available for cognitive recruitment. More generally, this study reveals how the active maintenance of cortical control over a continuous motor activity impacts on brain functioning and cognition.

## Introduction

Breathing belongs to the limited number of behaviors that can operate either under an automatic or a voluntary controlled mode, and the only one in this class of which the interruption poses an immediate vital threat. Schematically, the automatic mode is operated by brainstem respiratory pattern generators involving the Pre-Bötzinger complex and the parafacial/retrotrapezoid nuclei located in the medulla and their associated bulbospinal neurons [1], while the controlled mode depends on the activity of a large cortico-subcortical network including notably the anterior cingulate, supplementary-motor and insular cortices, as well as other regions [2]–[6].

Although this mapping between brainstem and automatic breathing on the one hand, and cortex and voluntary breathing on the other hand is central to our understanding of breathing, it does not inform us about the neural mechanisms at work, and about how these two modes of breathing interact. This issue, however, conveys major neuro-scientific and medical questions such as: how might the controlled mode network pilots the automatic structures when necessary? Is the controlled mode of breathing a conscious and voluntary reportable activity, or can it proceed unconsciously in conscious subjects, or even in non-conscious patients (e.g.: vegetative state patients)? Can this controlled mode of breathing be automatized? How is it coordinated with other cortically controlled motor processes which impact on breathing, such as speech production (segmentation, prosody) or playing a wind instrument?

This crucial issue is challenging because these two modes of breathing interact permanently in a complex and dynamical way. One way to disentangle them could consist in finding experimental or medical conditions in which awake and conscious subjects can be steadily engaged in each of these modes. To date, several functional neuroimaging and electrophysiological studies conducted in normal controls have used experimentally applied inspiratory constraints, - such as an inspiratory threshold loading -, to elicit a switch from automatic to cortically controlled breathing [7]–[13]. These works reliably demonstrated that the controlled mode of breathing is associated with cortical activation in many areas including premotor and bilateral insular cortices, and with decreased blood-oxygen-level dependent (BOLD) signal in regions of the DMN [14]. Interestingly, in line with the sustained nature of the respiratory-related cortical activity in response to a breathing difficulty [12], a recent study by Raux and colleagues [13] reported functional magnetic resonance imaging (fMRI) evidence that cortically-mediated breathing could itself be subject to automatization when using a continuous inspiratory load rather than an intermittent inspiratory load. Most of the cortico-subcortical areas associated with voluntary breathing showed a marked decrease of activation during continuous inspiratory loading as compared with intermittent inspiratory loading, in agreement with well-established motor skills automatization [15], [16]. A common limitation of the above studies is that experimental constraints used to manipulate breathing mode do not correspond to comfortable, ecological conditions for the subjects involved. Moreover, the impact of controlled and automatic breathing on subjective and objective cognitive measures has never been documented. For these reasons, it is considered extremely valuable to identify a stable, comfortable and regular breathing condition that demonstrates the implementation of the cortico-subcortical network associated with controlled breathing. One such very rare condition is congenital central hypoventilation syndrome (CCHS) in which the automatic control of breathing is irreversibly and massively impaired [17]. CCHS is an extremely rare disease (1/2–300000 births) that is characterized by alveolar hypoventilation and autonomic dysregulation [18]. CCHS patients usually have adequate breathing while awake, but significantly decreased breathing drive during sleep, including monotonous respiratory rates and diminished tidal volumes (shallow breathing). Patients therefore require mechanical ventilatory support (via nasal mask or tracheotomy) during sleep, to avoid life-threatening hypoxia consecutive to hypoventilation. Genetic studies have identified the paired-like homeobox 2B gene (PHOX2B) as the disease-defining gene [19]. Autopsy and structural MRI studies have identified subtle and disseminated white and grey matter impairments, affecting both supra-tentorial an infra-tentorial structures. Of particular interest, - given patients' physiological impairments -, is that several brainstem structures show structural abnormalities, including the locus coeruleus, parabrachial pons, caudal raphe nuclei, and lateral medulla (for a recent review see [20]). Several fMRI studies contributed by the Harper group explored brain responses of controls and of CCHS patients to various experimental conditions such as hypoxia, hyperoxia, cold pressor test, and forced expiratory loading [21]–[24]. Multiple brain regions responded inappropriately to ventilatory or blood pressure challenges, including forebrain, diencephalic, and brainstem related areas such as cerebellum.

Of importance regarding the issue of “resource competition” between the respiratory-related cortico-subcortical network and other cortical functions, anecdotal reports from parents of CCHS children suggest that mental concentration can deteriorate gas exchange (cyanosis during television watching, video gaming or scholarly exercise). Early publications on CCHS relayed this notion [25]–[27] that was subsequently challenged [28]. Indeed, Shea et al. showed that mental calculus and videogame playing increased ventilation both in CCHS in normal children. Yet these experiments did not take into account the emotional content of the test situation, and it was subsequently shown that video gaming in a neutral emotional environment induced hypoventilation in normal children [29]. Of note, this issue has not been addressed extensively in adults, although it has been shown that cortically-driven breathing is associated with deteriorated reaction times to an auditory stimulus [30]. Within this frame, adult CCHS patients exhibit a respiratory-related EEG activity during resting breathing [31] that resemble the potentials seen in normal subjects in response to inspiratory loading. It is currently unknown whether or not this respiratory-related cortical activity has an impact on operational and cognitive performances.

In the present study, we explored one patient affected with CCHS using a combination of behavioral and simultaneous electroencephalography (EEG) and fMRI brain-imaging measures both under spontaneous breathing (SB) and mechanical ventilation (MV) during wakefulness. We considered that this very rare medical condition could reveal how the active maintenance of cortical control over a regular motor activity impacts on broader cortical activity and function. More precisely, we designed this study in order to test our main hypothesis that during MV, resources used by the executive brain network would be freed up and hence made available for other cognitive purposes. Interestingly, when explaining the general objective of our experiment to the patient, she spontaneously reported that she had regularly switched to MV during her high-school years when needing to solve difficult problems or attend exams, with the subjective feeling of easier concentration and better cognitive performance as compared with SB. From our main hypothesis, we derived three predictions:

Prediction 1: Executive functions, - including sustained executive attention, working memory, executive control, and the richness of the stream of conscious thoughts-, should be more efficient during MV than during SB.

Prediction 2: Patterns of brain activity recorded during resting state should better match the normal DMN under MV than during SB.

Prediction 3: Patterns of functional brain connectivity should differ notably between SB and MV: a stronger correlation is predicted between the executive network and the brainstem during SB, whereas a stronger correlation within the DMN should be observed during MV than during SB.

This case-report falls within a long tradition of physiological and neuropsychological studies which demonstrate how focus on a single patient, if not necessarily representative of the concerned disease, can be decisive in enriching our understanding of impaired and normal physiology [32]–[35].

## Materials and Methods

### Patient

The patient is a 29-year-old woman. She was diagnosed with CCHS at the time of her birth. She carries a 9 alanine expansion mutation of the PHOX 2B gene. The main clinical manifestation of her condition pertains to ventilatory control, without any of the other frequent manifestations of the disease (in particular absence of Hirschprung disease and of cardiac rhythm anomalies). She does not increase ventilation and does not feel dyspnea when exposed to hypercapnia (in contrast to healthy subjects who reflexively hyperventilate and report respiratory discomfort in response to increased carbon dioxide levels), and she depends on mechanical ventilation during sleep. For this reason, she was tracheotomized at birth and until the age of 17, and has been ventilated non-invasively since. However, she does not exhibit hypoventilation during wakefulness, with arterial blood gases in room air within normal limits. Of note, this patient participated in another study that demonstrated that she displayed EEG cortical activity related to spontaneous ventilation [31]. The fMRI study was conducted in the frame of the RESPIRONDINE study (NCT01243697) into which the patient was enrolled. She had given her informed written consent to participate. Assistance Publique – Hôpitaux de Paris sponsored the study that was approved by the appropriate local legal and ethics authority (Comité de Protection des Personnes Ile-de-France 6, Pitié-Salpêtrière, Paris). Psychometric tests were part of the clinical follow up of the patient. Their study was approved by the institutional review board of the French Society for Respiratory Medicine (“Société de Pneumologie de Langue Française” reference number CEPRO2012-012). The patient gave her consent to anonymous use of her data for research purposes.

### Behavior

#### “Stream of consciousness” task

We adapted the task designed by [36]. The patient sat comfortably in a quiet dimly lit room. She was instructed: “During the next minutes, we ask you to keep your eyes closed and to avoid prolonged structured thinking, such as counting or singing. When you hear a beep, please indicate me first the intensity of ‘external awareness’ ongoing prior to the beep by reporting a number orally from 1 to 4, and then indicate me the intensity of ‘internal awareness’ ongoing prior to the beep by reporting a number orally from 1 to 4. ‘External’ is here defined as the perception of environmental sensory stimuli (e.g., auditory, visual, olfactory, or somesthetic). ‘Internal’ here refers to all environmental stimuli-independent thoughts (e.g., inner speech, autobiographical memories, or wandering thoughts).” This experiment was programmed with E-prime 1.1 software (Psychology Software Tools, Inc. Sharpsburg, PA).

#### Paced Auditory Serial Addition Test (PASAT)

The PASAT was delivered using a ‘ABBA’ design with four blocks in the following order: SB, MV, MV, SB. The patient used her own home mechanical ventilator and face mask. A pause was offered between blocks, and a longer pause of several minutes was used between the two transitions (SB to MV and MV to SB), so as to ensure that the patient was in a comfortable and steady state of respiration in each of the four blocks. The patient was tested with the 3 seconds version of the PASAT test [37] used to probe working memory and sustained executive attention (for a recent review see [38]). The patient was presented with a series of 60 single digit numbers with a 3 seconds inter-stimulus interval, and she was instructed to continuously sum aloud the last two digits, while the experimenter wrote her answers. In order to enable multiple evaluations, two versions of the task were available (tests A & B). The patient was tested with the following order: SB(A), MV(B), MV(A), SB(B). Each experimental block was preceded by a short training (training A, training B) made of 11 numbers.

### EEG recording & processing

**Figure 1:**
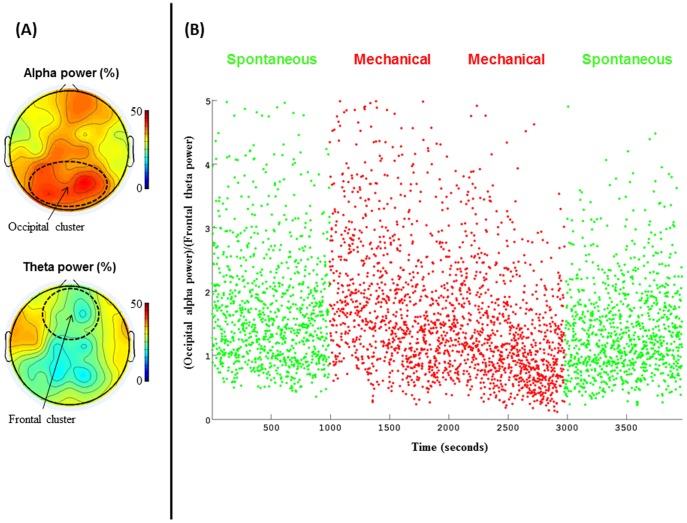
Stability of wakefulness under MV and SB indexed by EEG. (A). Scalp voltage topographies of alpha power (up) and theta (bottom) averaged across the 4 EEG-fMRI sessions reveal a typical pattern of wakefulness characterized by a high posterior alpha power and a low anterior mid-frontal power. (B). Dynamics of the (posterior alpha power)/(mid-frontal theta power) ration is plotted across time for the 4 EEG-fMRI sessions. This index of wakefulness was stable across the 4 sessions, and confirmed a stable level of wakefulness all along the fMRI experiments, with no difference between SB and MV.

Continuous EEG data was recorded at 5 kHz from 63 scalp sites (Easycap electrode cap) using MR-compatible amplifiers (BrainAmp MR and Brain Vision Recorder software; Brain Products). One additional electrode was placed on the collarbone to record the electrocardiogram (ECG). Impedances were kept under 15 kΩ. The EEG signal was corrected for MR related and for pulse artefacts (see SI). Then, for each time sample, we computed the ratio between relative alpha power averaged over occipital electrodes, and theta power averaged over frontal electrodes (see Figure 1A). This alpha/theta EEG power ratio is a common measure of vigilance [39]–[41].

The data were subsequently downsampled to 250 Hz and re-referenced to a common average reference. Visual inspection of the signal checked for the absence of residual obvious artefacts. Time-frequency (TF) analysis was then computed with a Morlet wavelets approach in Fieldtrip (http://www.ru.nl/fcdonders/fieldtrip). TF was computed for frequency from 0.5 to 15 Hz, with a frequency step of 0.5 Hz, a time step of 0.5 sec, and a wavelet width of 4 cycles. The (posterior alpha)/(theta midfrontal) ratio was computed on the normalized power (normalization across the 0.5–15 Hz frequency range), using the following electrodes (Easycap electrode cap numbers): occipital region of interest (ROI) [9 10 19 20 31 37 38 45 46 64], and mid-frontal ROI [1 2 3 4 17 33 34 39 40 63]. Statistical comparisons across MV and SB were performed using the non-parametric Wilcoxon rank-sum test.

### Magnetic resonance (MR) imaging

#### MR acquisition protocol

The MR protocol was carried out with a 3T whole-body system (Siemens, Erlangen, Germany) at the Center for Magnetic Resonance Research (CENIR), Institute of the Brain and the Spinal Cord (ICM), Paris. The functional images were acquired by T2*-weighted fast echo planar imaging (EPI; flip angle = 90°, echo time = 30 ms, repetition time = 2.26 s) from 36 interleaved axial slices (Field Of View = 100×100×36, gap = 0.3 mm) with a 2×2×2 mm3 voxel size for the resting state. The resting-state fMRI experiment consisted of one 10-minute run in which the patient was instructed to relax with her eyes closed, without falling asleep. Each run consisted of 200 EPI volumes. Subsequently, a high-resolution structural volume was acquired using a 3D magnetization prepared rapid gradient echo (MP-RAGE) sequence (144 sagittal images; thickness 1 mm; FOV 256×256 mm2; matrix size 256×256). Immediately before each of the four “resting state” blocks, the patient received the following instructions: “please, keep your eyes closed, stay awake during the entire block, and try to let your mind wander to any particular thought”. As for the behavioural tests, we also used an ‘ABBA’ design with the following order: SB, MV, MV and SB. After each of the 4 scanning blocks a brief interview checked the absence of breathing discomfort, the absence of subjective report of drowsiness, and the effective engagement in the “mind-wandering” state. SpO2 was measured during the fMRI experiment.

#### fMRI data processing & analysis

A correction was applied to reduce physiological noise (see SI) using a retrospective estimation and correction of respiration and heart beat [42].

#### General linear model

A general linear model was created, which included the 4 sessions, each modeled by the canonical hemodynamic response function and its first-order time derivative, and 6 individual motion parameters to capture remaining signal variations due to head movements. T-test based contrasts of interests were then defined in SPM8. We reported significant results at the cluster-level (all SPM results are reported with the following significance threshold: p FWE-correction ≤0.05). For significant clusters we also report the peak-level T value as well as the cluster volume (number of voxels).

#### Functional connectivity assessed with an fMRI or EEG ROI based analysis

Three ROIs were selected: (1) the precuneus which is major hub of normal DMN, (2) the brainstem which is in charge of automatic control of breathing, and (3) auditory cortex as a control region not primarily implicated in the control of breathing. The precuneus ROI (seed region 1) was defined using A canonical template (automatic anatomical labeling from WFU PickAtlas toolbox of SPM, http://fmri.wfubmc.edu/software/PickAtlas), from which the time course was extracted. We looked at the brain areas that showed a stronger correlation of their time course with the seed region during MV than during SB. We then used a brainstem ROI (seed region 2) manually defined as corresponding to the medulla oblongata: it was delineated from the pons by a horizontal boundary, and occupying a region extending distance ventrally to the estimated boundary with the spinal cord. The third control ROI (seed region 3) was defined using the Automated Anatomical Labelling atlas in regions of the left and right hemisphere considered to closely represent the auditory cortex, namely Herschl's gyrus [43]. The same approach was used to compute correlation of BOLD signal with the posterior EEG alpha-power time series.

#### Functional connectivity assessed with a spatial ICA based analysis

We used a spatial independent component analysis (ICA) approach to extract group representative functional large-scale networks across the whole brain (see Materials & Methods S1 and [44] for details).

## Results

### Behavior

The patient was tested in three tasks exploring various aspects of executive functions as well as the reportability of conscious contents.

#### “Stream of consciousness” test

This task aimed at comparing both the type and the subjective intensity of current conscious contents during the two modes of breathing. As previously showed by [36] internally and externally oriented contents were anti-correlated (correlation coefficient = −0.21; p-value = 0.1). The small number of trials (N = 34) probably explains the weak significance of this anti-correlation. During MV we observed higher values of both internal and external awareness contents as compared with SB (both p values = 0.02 in Wald-Wolfowitz runs tests). This pattern, affecting both external and internal reportable contents, is compatible with our prediction that MV would act by “releasing” attentional and executive resources available for other cognitive activities and contents.

#### Paced Auditory Serial Addition Test (PASAT)

The patient was engaged in this classical test probing both working memory and sustained executive attention. Error rate remained constant across the four experimental blocks (43/60 & 43/60 for the two SB sessions, and 45/60 & 43/60 for the two MV sessions; Chi 2 value = 0.8; p = 0.8). However, a post-hoc analysis revealed that when analyzing the length of chunks of consecutive correct answers, we observed a clear advantage for the MV condition: sequences ranged from 1 to 6 under SB (mean = 3.3), and from 1 to 16 under MV (mean = 4.4). In particular a non-parametric ranking test showed that sequences superior to 5 consecutive correct trials were longer in the MV condition than in the SB condition (p = 0.02 in Mann-Whitney U test).

Taken together, these results support our initial prediction by showing an impact of cortically-controlled breathing on several facets of executive functions. As compared with performances obtained during SB, the patient showed an improvement of sustained attention during MV, and her conscious reports revealed more intense contents during MV than during SB.

### EEG-fMRI sessions

After each of the four scanning blocks patient debriefings did not reveal any breathing discomfort, or any subjective report of drowsiness. She confirmed having been engaged in the “mind-wandering” state in each of the 4 blocks. Pulse oximetry saturation (SpO2) ranged from 93% to 100%, with no significant difference across blocks.

### EEG

Recording EEG during fMRI acquisition was motivated by the need for an objective physiological marker of vigilance during the resting state sessions, in particular to ensure the absence of drowsiness under mechanical breathing. Even if the patient did not report any drowsiness during the four blocks, subtle variations in vigilance may contribute to differences in fMRI patterns. An EEG index of vigilance (ratio of occipital alpha/frontal theta; see M&M and Figure 1A) did not reveal any difference between the four blocks (see Figure 1B; Wilcoxon rank sum test rank p value = 0.33). In order to better assess the validity of our single subject EEG-fMRI measures, we also analyzed which brain areas (fMRI signal) were correlated with the alpha-power averaged across posterior electrodes (EEG signal). While no positive correlation was observed, this analysis revealed a large fronto-parietal network negatively correlated with alpha-power (see Figure S1 and Table S1), as previously reported by [45] and many other teams during wakefulness (see [46] for a review). The results hence confirmed that levels of vigilance were consistent across the four blocks tested.

### fMRI

**Figure 2:**
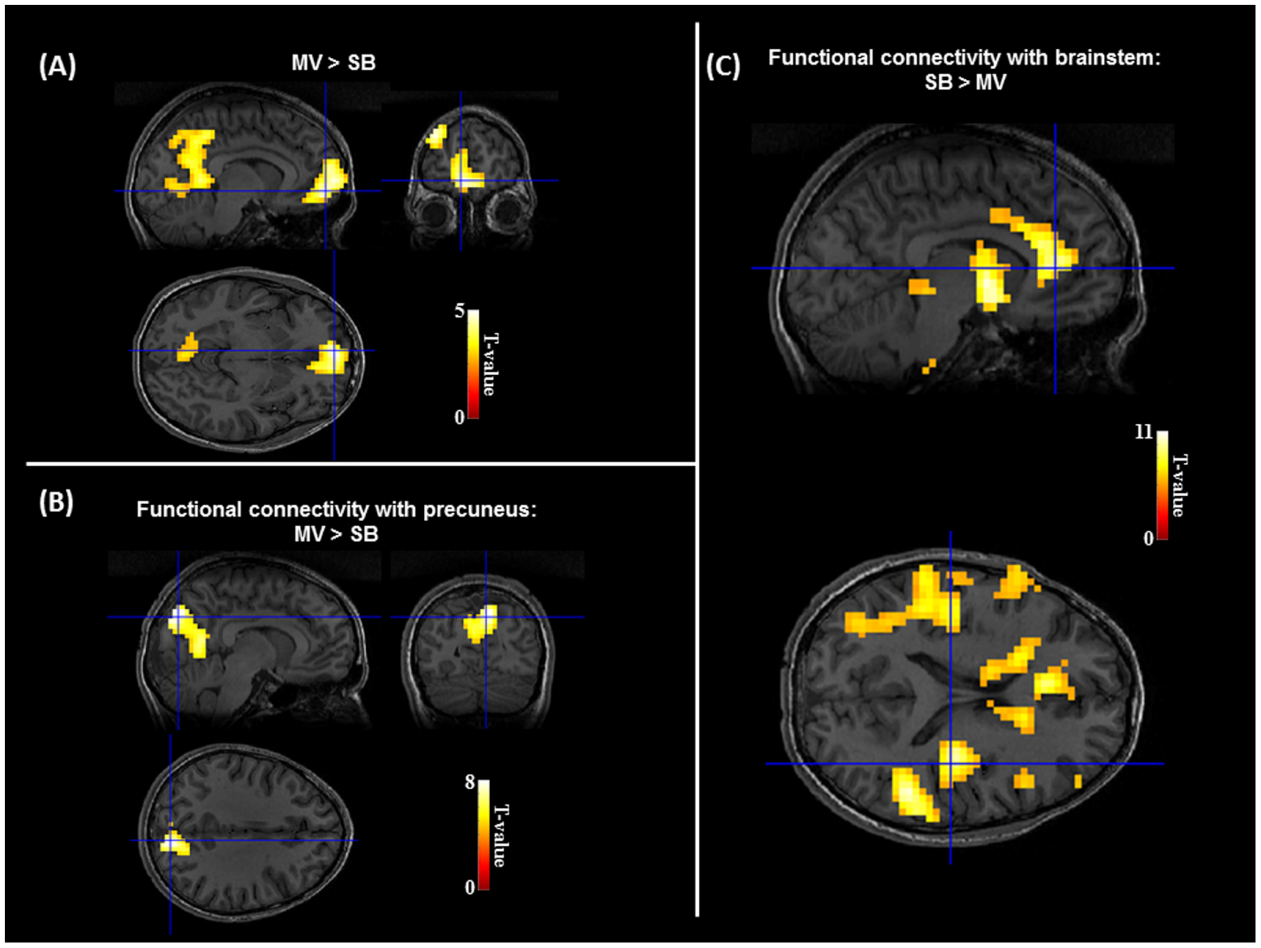
Restoration of DMN under mechanical ventilation. (A). Comparison of BOLD signal between MV and SB revealed a specific increase of activation in the default-mode network associated in awake controls in introspection and self-consciousness. No significant result was observed in the opposite contrast. See Table 1 for detailed fMRI results. (B&C). Functional connectivity assessed with a hypothesis-driven approach revealed a larger correlation with precuneus activity in posterior mesial areas during MV than during SB (B), and a larger correlation between brainstem activity and a large anterior cortico-subcortical network during SB than during MV (C). This large network resembles the executive attention network.

Brain areas more activated during MV than during SB corresponded to the typical DMN including parietal-occipital mesial areas (precuneus and posterior cingulate cortices) and anterior mesial-frontal areas (see Figure 2A and Table 1). Interestingly, no brain area showed any increase of activation in the reverse contrast (SB>MV).

**Table 1:** Synthesis of fMRI results.

Anatomical localization of cluster	Coordinates [x,y,z]			T score	Number of voxels
**Activation: MV>SB**					
Medial orbito-frontal cortex (BA11)	−7	59	−9	4,43	380
Middle frontal gyrus (BA8)	−51	19	43	4,35	292
Calcarine sulcus	−11	−51	4	4,19	473
Precuneus, posterior cingulate cortex	−8	−55	15	3,75	473
**Connectivity Precuneus: MV>SB**					
Cuneus (BA19)	11	−82	35	7,95	426
Lingual gyrus (BA19)	11	−54	−3	6,63	426
Calcarine sulcus	−15	−60	6	6,39	426
Middle frontal gyrus (BA6)	50	−3	52	5,65	42
**Connectivity Precuneus: SB>MV**					
Thalamus, tectum	−7	−29	4	7,33	319
Caudate tail	−17	−15	22	6,1	
Cerebellum (Crus 1)	35	−53	−32	6,07	39
Brainstem	8	−39	−3552	5,94	22
Cerebellum (lobule 7b)	24	−78	−49	5,82	54
Superior frontal gyrus	24	25	62	5,7	33
Inferior orbito-frontal cortex (BA11)	−31	36	−12	5,36	36
**Connectivity Brainstem: SB>MV**					
Pallidum	−9	1	−6	10,69	1347
Anterior cingulate cortex (BA32)	−6	42	7	9,41	1347
Posterior insula	−39	−22	14	9,36	1347
Posterior insula	36	−17	14	10,51	99
Anterior insula	38	0	5	9,44	343
Brainstem	−3	−31	−50	8,08	13
Tectum	−5	−31	−7	6,89	29
Precentral gyrus, inferior frontal gyrus	61	8	20	6,36	10
Pars triagularis frontalis	−49	41	7	6,12	17
Anterior insula	−30	27	−3	6,03	11
**Connectivity Brainstem: SB>MV**					
Cerebellum (lobule 9)	−14	−43	−51	7,72	32

**Figure 3:**
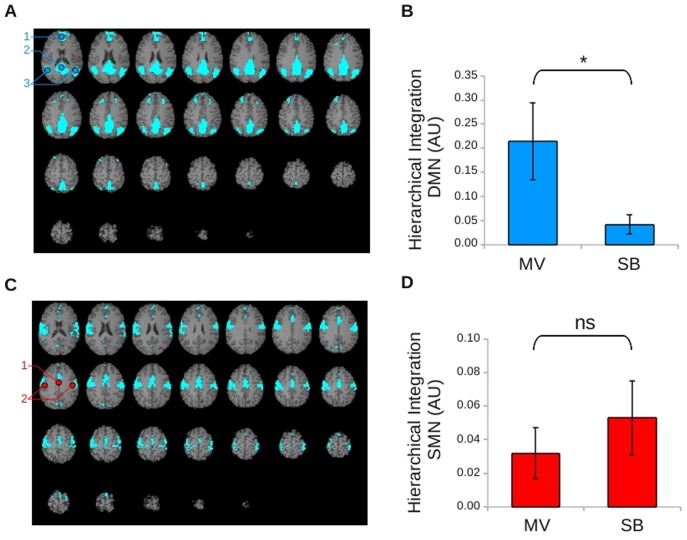
Functional MR connectivity assessed with an ICA method. (A).Default mode network (DMN): bilateral medial prefrontal and anterior cingulate cortices (1), bilateral precuneus (2), bilateral superior parietal cortices (3). (B).Mean values ± standard deviation of DMN integrations for the mechanical (MV) and the spontaneous (SB) breathing blocks of resting state. The asterisk indicates a significant difference between conditions. (C). Sensorimotor network (SMN): bilateral supplementary motor area (1) and bilateral sensorimotor cortices (2). (D).Mean values ± standard deviation of SMN integrations for the mechanical (MV) and the spontaneous (SB) breathing blocks of resting state. NS  =  non-significant difference between conditions; AU  =  arbitrary unit.

In order to better understand the mechanisms at work during the two breathing conditions, we performed complementary analyses exploring patterns of functional connectivity. We defined a ROI in the precuneus (seed region 1) and looked for brain areas showing a stronger correlation of their time course with the seed region during MV than during SB. Only the posterior mesial structures, belonging to the normal DMN showed such a profile (see Figure 3B), whereas a set of subcortical and frontal structures showed a reverse profile (SB>MV). These last structures included superior frontal gyrus and inferior orbito-frontal cortex, as well as thalami and caudate nuclei, cerebellum and brainstem regions (see Table 1). This ROI analysis confirms that a major hub of normal DMN, - the precuneus -, varies its functional connectivity in relation with the respiratory condition. Whereas precuneus was connected with other postero-mesial normal DMN structures under MV, it showed an unusual connectivity during SB with an anterior network suggestive of a frontal-subcortical control of brainstem breathing structures.

We then used a brainstem ROI (seed region 2), which showed a stronger functional connectivity with a cortico-subcortical anterior network during SB than during MV (see Figure 2C). This network included several frontal lobe and anterior cingulate areas, as well as insular regions and the caudate nuclei, which have been previously implicated in effortful breathing [7]–[9], [13]. A control ROI (seed region 3) defined in the primary auditory cortex did not show any modulation of functional connectivity when comparing SB and MV blocks.

Finally, we used a non-arbitrary method based on spatial independent component analysis to quantify functional connectivity within brain-scale networks. Hierarchical integration was significantly larger within the DMN during MV than during SB (p = 0.997 considered as significant using Bayesian statistics with p>0.909 as the threshold of significance; see methods and Figure 3A & 3B). A non-significant trend in the opposite comparison (SB>MV) was observed within the SMN (p = 0.818, see Figure 3C & 3D).

## Discussion

The goal of this study was to take advantage of a very rare syndrome, - characterized by a massive impairment of the automatic control of breathing-, to explore how the active maintenance of cortical control over an enduring motor activity impacts on cortical activity and function. In a CCHS patient, we tested the general hypothesis that the executive brain network, whose intervention is required for controlling breathing during SB, should be released and made more available for other cognitive purposes during MV. We will now discuss these results by addressing each of our three empirical predictions.

### Prediction 1: Better executive functioning during MV than during SB

We tested the patient in two behavioral experiments. First, the PASAT revealed a significant increase in the length of sequences of correct responses during MV as compared to SB. The ‘ABBA’ design prevented a systematic confound of breathing condition with time, and this result therefore confirms the prediction that working memory and sustained executive attention were improved during MV. We also observed a general increase in the subjective intensity of spontaneous conscious contents under MV, as compared to SB, during a resting state experiment. We regret the absence of ‘ABBA’ design for this last experiment, but this was due to a schedule constraint of the patient. One may have expected internally-related thoughts to be more frequent than externally-related ones during MV by releasing executive resources from breathing control. The fact that we did not observe such a trend may call for more subtle probing of conscious thoughts, to check specifically if breathing related thoughts decreased during MV. Note also that even if we had tried to clearly categorize sensory-motor and somesthesic thoughts as being related to the external world, breathing may have a special status, intermediate between our external and internal categories. A complementary way to test this possibility would consist in replicating this experiment during an fMRI scan, and to check the respective correlates of internal and external thoughts under SB and MV. Taken together, both objective (PASAT) and subjective behavioral measures tended to confirm our prediction that the engagement of executive functions in various cognitive tasks increased under MV in comparison with SB. In addition to our main goal, this result may prove relevant to explain the mild cognitive disabilities presented by most CCHS patients [47], [48]. If this result was confirmed on a larger cohort of patients and in a larger set of tasks, one may consider prescribing MV in these patients for cognitive purposes such as in academic study, in addition to preventing sleep hypoventilation.

### Prediction 2: Restoration of DMN networks during MV as compared to SB

The most impressive result of this study is the specific increase of DMN activity during MV as compared to SB. We checked the absence of several potential confounds and caveats. First, the ‘ABBA’ design we used discarded a possible confound with time, which may be interpreted in terms of habituation. Second, we ensured the absence of a confound with drowsiness under MV during which the patient may have relaxed and decreased her level of vigilance. Verifying the level of vigilance was particularly important to check because CCHS patients often associate MV with a secure feeling of riskless sleep. For this purpose, the combination of clear instructions, of post-block subjective debriefing and most importantly of continuous EEG recording during fMRI acquisition permitted us to discard any difference in level of wakefulness between MV and SB. Third, we checked that raw parameters of breathing (blood oxygenation, respiratory rate and subjective breathing comfort) were comparable in both conditions. All these controls suggest that a correct interpretation of this fMRI result fits with our prediction: under MV the anterior executive network activity is released from breathing, and is then available to contribute to the normal neuronal signature of awake resting state. This observation is reminiscent of the decrease in DMN activity that occurs shortly after the application of an inspiratory load in normal controls [13]. It would be very valuable to verify whether this result could apply on a larger group of CCHS patients, and to combine it with subjective measures of conscious content in order to more precisely describe the nature of cognitive processes at work in these patients during MV. The raw debriefing we used only confirmed that the patient really engaged in mind-wandering in both breathing conditions, but more precise measures would certainly be valuable. Finally, the absence of a significant result in the reverse contrast (SB>MV) suggests that frontal areas presumably implicated in the control of breathing during SB were engaged in other tasks during MV with a globally similar level of neural activity. Actually, when we decreased the statistical thresholds (p<0.1, 30 voxels), activated regions began to include frontal areas including dorsolateral prefrontal and anterior cingulate cortices, striatum, as well as a right parietal region. This last finding further strengthens the utility of functional connectivity measures.

### Prediction 3: Functional connectivity patterns of brain activity differ during SB and MV

In order to better explore the previous prediction, we used two complementary approaches to evaluate how the breathing modality affected brain-scale functional connectivity in this patient. Using a hypothesis-driven seed approach, we first showed that one hub of the DMN, - namely the precuneus region -, did increase its functional connectivity with other posterior mesial areas under MV as compared with SB. One may have expected an increase of functional connectivity with anterior regions of the DMN. Actually, when statistical thresholds were decreased (p<0.1; 30 voxels), we were able to identify such frontal areas. Interestingly, the reverse contrast (SB>MV) revealed an increased functional connectivity of precuneus with frontal and subcortical regions suggestive of a motor control network. Using a data-driven approach we showed that the DMN network was more strongly integrated (as measured by the hierarchical integration index) under MV than under SB. These two functional connectivity results added coherence to the larger BOLD activation we observed in DMN hubs during MV than during SB (see Prediction 2). The last result we obtained confirmed the existence of a stronger correlation between the executive network and the brainstem during SB in comparison with MV. It supports previous reports of co-activation of cortical and brainstem areas in response to breathing loads in normal subjects [7], [8], [13], [49], and of a recent transcranial magnetic stimulation (TMS) study showing that SMA modulates the cortico-spinal pathway piloting brainstem structures [50].

Overall, this study revealed some important cues related both to the neural mechanism subtending cortical control of brainstem activity during breathing, and to the consequences of such control in terms of functional connectivity and of cognitive processing. These results strengthen the plausibility of our general hypothesis according to which MV would act as freeing up executive resources available for other cognitive purposes (e.g.: introspection during resting state; executive control during the PASAT). A full demonstration of this hypothesis would require additional works including: a replication of these results in a larger population of CCHS patients taking into account the various possible genotypes (e.g.: alanine expansion lengths, various PHOX2B gene mutations), as well as more causal evidence such as for instance the perturbation of SB in CCHS patients in response to TMS inhibition of executive control network. Among the many issues that remain to be addressed, two are considered to be of particular importance: consciousness of cortically-driven breathing, and the value of our findings to the management of CCHS.

The brainstem-driven versus cortex-driven dichotomy of breathing modes is frequently associated with another dichotomy originating from the field of cognitive psychology and information processing: unconscious automatic versus conscious voluntary types of neural processing. However, if many arguments support the unconscious nature of brainstem reflexive breathing, the conscious and voluntary nature of cortex-driven controlled mode of breathing is less evident. In our study, the patient did not report any feeling of voluntary control of breathing (agentivity) during SB. However, we did show that SB was associated with a controlled mode of breathing correlated with the activity of an extended anterior cortical network in this patient. This is coherent with previous observations in the same patient having evidenced the presence of pre-inspiratory potentials during SB, a phenomenon normally absent in healthy subjects [31]. Therefore the contribution of an extended cortical network to breathing control does not guarantee that this form of control is a consciously reportable process. This intriguing possibility of a non-conscious form of cortical control of breathing is even more compelling given that it seems to require cognitive resources typically associated with consciousness such as working memory and dynamic executive control [51]. Interestingly, current cognitive neuroscience studies suggest that some complex cognitive processes of external stimuli (e.g.: dynamic regulation of control; metacognitive effects) may operate unconsciously but still require conscious processing of the stimuli to occur. For instance, in the field of visual perception many studies reported that only consciously visible stimuli can elicit sustained strategical changes [52], [53], or dynamic regulation of executive control such as the Gratton effect ([54], [55], but see [56]). In sharp contrast, when the very same stimuli are presented under conditions of invisibility (e.g.: subliminal stimuli using visual masking), they are still processed unconsciously but without eliciting such complex effects [51]. However, in these studies most subjects don't have any introspection or conscious agentivity of these strategical changes that were present only when they were conscious of the stimuli [57], [58]. This set of findings may be relevant for the case of breathing: it is possible that the cortically-controlled breathing (or, more generally, a cortical contribution to the overall drive to breathe) would require the subject to be awake and conscious, even if the subject was not necessarily conscious of breathing itself. One consequence of this hypothesis would be that no cortical control of breathing should be observed in awake but non conscious patients such as vegetative state patients. Interestingly, the recent fMRI study by Raux et al. [13] suggests that cortically-controlled breathing may itself be automatized. Future studies should combine functional neuroimaging measures with fine subjective reports under various breathing conditions in order to better explore this complex issue.

We will conclude by briefly discussing the value of our finding in regard to the pathophysiology of CCHS. Obviously, a single-patient report can only contribute at opening new research perspectives. Still, if our findings were to be replicated on additional CCHS patients they may suggest that MV could be useful for enhancing cognition during wakefulness, in addition to its current use to prevent hypoxia and death during sleep. Indeed, both spontaneous subjective reports of this patient, as well as our fMRI and behavioral results suggest that MV improves executive function. Given the frequent mild cognitive disabilities of CCHS patients from early childhood, prescription of MV during school time or similar cognitively oriented activities may be particularly relevant to optimize their learning processes, and to improve durably their intellectual abilities.

## Supporting Information

Figure S1 **Areas showing a negative correlation with posterior alpha power (T-scores maps).**
(TIF)

Table S1 **Areas showing a negative correlation with posterior alpha power.**
(DOCX)

Materials and Methods S1 **Behavior, EEG and fMRI supplementary methods.**
(DOCX)
