# Supplementary figures and images for: The Cerebral Cost of Breathing: An fMRI Case-Study in Congenital Central Hypoventilation Syndrome
Source: PLoS One. 2014 Sep 30;9(9):e107850. doi: 10.1371/journal.pone.0107850 (PMC4182437; doi:10.1371/journal.pone.0107850)

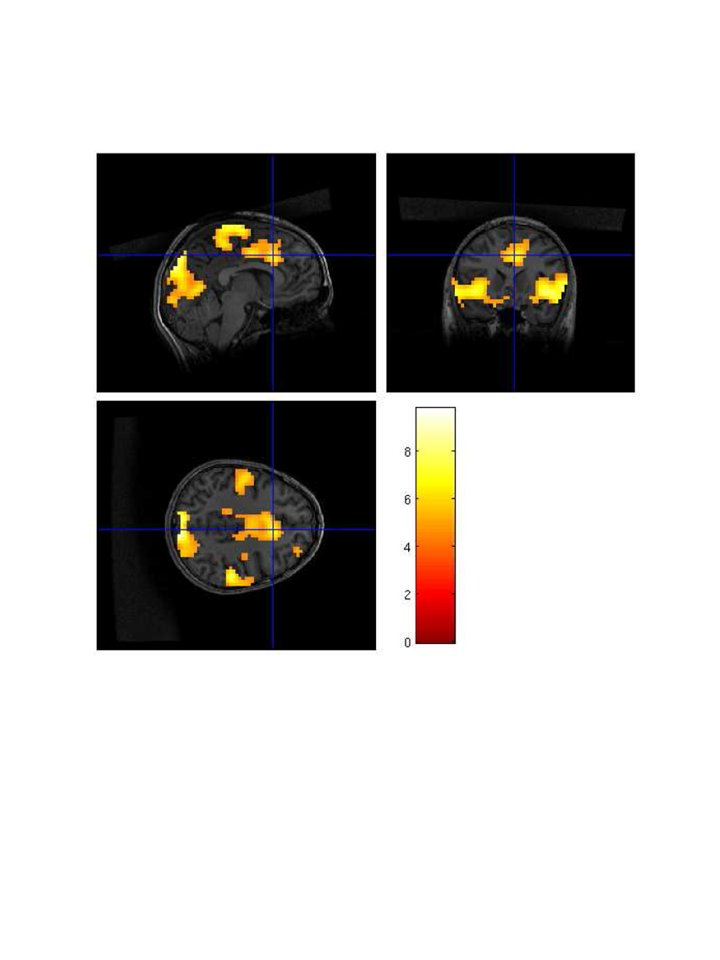

Supplement: Figure S1 — Areas showing a negative correlation with posterior alpha power (T-scores maps). (TIF) [file pone.0107850.s001.tif]
