# Supplementary material for: The Cerebral Cost of Breathing: An fMRI Case-Study in Congenital Central Hypoventilation Syndrome
Source: PLoS One. 2014 Sep 30;9(9):e107850. doi: 10.1371/journal.pone.0107850 (PMC4182437; doi:10.1371/journal.pone.0107850)
Supplement: Table S1 — Areas showing a negative correlation with posterior alpha power. (DOCX) [file pone.0107850.s002.docx]

**SI Table:** **Areas showing a negative correlation with posterior alpha power**

**SOM Figure: Areas showing a negative correlation with posterior alpha power**

**References**

Esposito, F., T. Scarabino, et al. (2005). Independent component analysis of fMRI group studies by self-organizing clustering. Neuroimage. United States. **25:** 193-205.

Marrelec, G., P. Bellec, et al. (2008). Regions, systems, and the brain: hierarchical measures of functional integration in fMRI. Med Image Anal. Netherlands. **12:** 484-96.

Marrelec, G., A. Krainik, et al. (2006). Partial correlation for functional brain interactivity investigation in functional MRI. Neuroimage. United States. **32:** 228-37.

Perlbarg, V. and G. Marrelec (2008). "Contribution of exploratory methods to the investigation of extended large-scale brain networks in functional MRI: methodologies, results, and challenges." Int J Biomed Imaging **2008**: 218519.
