# Supplementary material for: The Cerebral Cost of Breathing: An fMRI Case-Study in Congenital Central Hypoventilation Syndrome
Source: PLoS One. 2014 Sep 30;9(9):e107850. doi: 10.1371/journal.pone.0107850 (PMC4182437; doi:10.1371/journal.pone.0107850)
Supplement: Materials and Methods S1 — Behavior, EEG and fMRI supplementary methods. (DOCX) [file pone.0107850.s003.docx]

**Materials & Methods S1:**

***.Behavior:***

“Stream of consciousness task”: Each experimental block lasted for 7 minutes, with beeps randomly spaced by 10 to 24 seconds. Before each the two experimental blocks (SB and MV), the patient was engaged in a short training block made of 10 trials sharing the same temporal structure. The patient was first tested during SB and then during MV. We initially planned to run a second set of blocks with the reverse order (MV, SB), in order to obtain a full ‘ABBA’ design. Unfortunately, this second stage of testing had to be cancelled due to schedule constraints of the patient.

***.EEG:***

Gradient and pulse artifacts were first marked using an in-house software (http://wiki.cenir.org/doku.php/datahandler). Artifacts were detected by correlation with a template automatically or manually defined. We used the raw signal of channel FC2 for gradient artifacts, and the raw ECG signal filtered between 0.5 and 10 Hz for pulse artifacts. We used eeglab v9.0.8.6b (<http://sccn.ucsd.edu/eeglab>) and the fmrib plug-in (<http://fsl.fmrib.ox.ac.uk/eeglab/fmribplugin/>) for gradient and pulse artifact subtraction.

***.fMRI processing:***

Artefact correction: K-space functional data were processed to reduce physiological noise using a retrospective estimation and correction of respiration and heart-beat. The first two and the last volumes were discarded for signal stabilization. A temporal cut-off (cutoff frequency 4.16 × 10^−3^ Hz) was applied to the functional data to filter out subject-specific low-frequency drifts of the signal. Data were then corrected for subject’s motion, with the first volume of each run as a reference, and resliced using 4^th^ degree B-Spline interpolation with the SPM8 software. Finally, the same software was used to smooth the images with an isotropic Gaussian spatial filter of full-width-at-half-maximum 5 mm. Anatomical images were normalized to the standard brain template defined by the Montreal Neurological 152-brains average.

Functional connectivity assessed with a spatial ICA based analysis: We anatomically localized the ROIs of each network on the corresponding spatial maps. Spatial ICA analysis was carried out [[1](#_ENREF_1)]. First, the 20 spatial components explaining the most variance were extracted using an infomax ICA algorithm. These components were scaled to z-scores and registered to the MNI standard space using nonlinear spatial transformations as implemented in SPM8. The second step consisted of clustering independent components based on their spatial similarity [[2](#_ENREF_2)]. Finally, partitioning of the hierarchy was automatically performed, using criteria optimizing both the unicity and the representativity of each extracted class. For each of the resulting condition-representative classes (MV and SB), a fixed-effect map of t-scores was computed for the two conditions, thresholded at p ≤ 0.05 uncorrected for multiple comparisons. Within this set of condition-representative maps exhibiting spatially structured functional processes and noise processes, we verified the maps which exhibited a spatial organization distributed into areas of DMN and SMN (sensory motor network). We then applied a measure deriving from entropy, - hierarchical integration -, which is aimed at quantifying information exchanges within networks [[3](#_ENREF_3)]. Hierarchical integration derives from functional connectivity and summarizes correlation coefficients between ROI time series in one single number. For instance, when all correlation coefficients are equal to zero (i.e., there is no functional connectivity), integration is also equal to zero; otherwise, it is positive. The integration of a given network can be decomposed into the sum of the integration measured within each component (DMN: precuneus, parietal, anterior cingulate; SMN: supplementary motor area (SMA), lateral premotor cortex, somatosensory cortex). We studied the total integration within each network. Values of integration were inferred using a Bayesian numerical sampling scheme that approximated the posterior distribution of the parameters of interest in a group analysis [[4](#_ENREF_4)]. A mean value and a standard deviation for each value of integration were then computed. A total of 5000 integration values were computed from the sampling scheme, from which statistics can be obtained. We tested that the posterior probability p(A|y) of the assertion that “the SB condition has lower integration than MV condition” (and vice-versa for the SMN) related to any pair of integration measure in each network. The validity of the assertion [[5](#_ENREF_5)] measured in decibels (dB), related to the ratio of the probability that A to be true to the probability that A to be false, in a base 10 logarithmic scale. According to this measure, two values of integration will be said significantly different when |e(A|y)| > 10 dB, which corresponds to a probability of A being true p(A|y) > 0.909. A significant positive value of evidence shows that the assertion A is true, whereas a significant negative value shows that the complementary assertion Ā has to be considered as true.

**References**

1. Perlbarg, V. and G. Marrelec, *Contribution of exploratory methods to the investigation of extended large-scale brain networks in functional MRI: methodologies, results, and challenges.* Int J Biomed Imaging, 2008. **2008**: p. 218519.

2. Esposito, F., et al., *Independent component analysis of fMRI group studies by self-organizing clustering*, in *Neuroimage*2005: United States. p. 193-205.

3. Marrelec, G., et al., *Regions, systems, and the brain: hierarchical measures of functional integration in fMRI*, in *Med Image Anal*2008: Netherlands. p. 484-96.

4. Marrelec, G., et al., *Partial correlation for functional brain interactivity investigation in functional MRI*, in *Neuroimage*2006: United States. p. 228-37.

5. Jaynes, E.T., *Probability Theory: The Logic of Science. Vol. I – Principles and Elementary Applications*2003, Cambridge: Cambridge University Press.
